# First prospective clinical evaluation of feasibility and patient acceptance of magnetic resonance-guided radiotherapy in Germany

**DOI:** 10.1007/s00066-020-01578-z

**Published:** 2020-01-30

**Authors:** Sebastian Klüter, Sonja Katayama, C. Katharina Spindeldreier, Stefan A. Koerber, Gerald Major, Markus Alber, Sati Akbaba, Jürgen Debus, Juliane Hörner-Rieber

**Affiliations:** 1 Department of Radiation Oncology, Heidelberg University Hospital, Im Neuenheimer Feld 400, 69120 Heidelberg, Germany; 2 Heidelberg Institute of Radiation Oncology (HIRO), Heidelberg, Germany; 3 National Center for Tumor diseases (NCT), Heidelberg, Germany; 4 Department of Radiation Oncology, Heidelberg University Hospital, Heidelberg Ion-Beam Therapy Center (HIT), Heidelberg, Germany; 5 Clinical Cooperation Unit Radiation Oncology, German Cancer Research Center (DKFZ), Heidelberg, Germany

**Keywords:** MR-guided, MR-Linac, Implementation, Gating, Patient acceptance, Patient-reported outcomes

## Abstract

**Purpose:**

Magnetic resonance-guided radiotherapy (MRgRT) has recently been introduced in our institution. As MRgRT requires high patient compliance compared to conventional techniques and can be associated with prolonged treatment times, feasibility and patient tolerance were prospectively assessed using patient-reported outcome questionnaires (PRO-Q).

**Materials and methods:**

Forty-three patients were enrolled in a prospective observational study and treated with MRgRT on a low-field hybrid Magnetic Resonance Linear Accelerator system (MR-Linac) between April 2018 and April 2019. For assistance in gated breath-hold delivery using cine-MRI, a video feedback system was installed. PRO-Qs consisted of questions on MR-related complaints and also assessed aspects of active patient participation.

**Results:**

The most commonly treated anatomic sites were nodal metastases and liver lesions. The mean treatment time was 34 min with a mean beam-on time of 2:17 min. Gated stereotactic body radiotherapy (SBRT) was applied in 47% of all patients. Overall, patients scored MRgRT as positive or at least tolerable in the PRO‑Q. Almost two thirds of patients (65%) complained about at least one item of the PRO‑Q (score ≥4), mainly concerning coldness, paresthesia, and uncomfortable positioning. All patients reported high levels of satisfaction with their active role using the video feedback system in breath-hold delivery.

**Conclusion:**

MRgRT was successfully implemented in our clinic and well tolerated by all patients, despite MR-related complaints and complaints about uncomfortable immobilization. Prospective clinical studies are in development for further evaluation of MRgRT and for quantification of the benefit of MR-guided on-table adaptive radiotherapy.

## Introduction

Image-guided radiotherapy (IGRT) allows for daily monitoring of patient and tumor positioning and, to some extent, immediate detection of alterations in tumor volume and patient anatomy [1–3]. Currently, image guidance is mainly based on kilovoltage or megavoltage computed tomography (CT) imaging as the standard of care, which is routinely incorporated in most modern radiotherapy units. However, onboard CT imaging only offers poor soft tissue contrast and hence primarily enables image guidance based on bony anatomy [4].

Magnetic resonance (MR) imaging, with its superior soft-tissue contrast, facilitates enhanced differentiation between cancerous and healthy tissue as well as functional assessment of treatment response [5, 6]. Given the technical challenges in integrating MR imaging (MRI) into a linear accelerator, first studies on MR-guided radiotherapy (MRgRT) focused on offline solutions and reported efficacy and feasibility for this technique [7–10].

Recently, devices integrating an MRI scanner with a treatment delivery system have become clinically available [11–15]. These new hybrid systems for MRgRT do not only offer three-dimensional MRI for soft tissue target and organ at risk visualization, but also allow for continuous cine-MRI before as well as during treatment [12, 13]. Cine-MRI during treatment enables advanced motion management based on real-time soft tissue anatomic feedback such as treatment beam gating [11, 16, 17]. This eliminates the need for invasive implantation of fiducial markers as well as the application of internal target volumes (ITV) in order to account for intrafractional motion, and thereby offers the potential for margin reduction and hence a lower risk of ensuing toxicity [18].

Online MRgRT was implemented at our institution in April 2018 using the MRIdian Linac® system (ViewRay Inc.; Oakwood, USA), which combines a 0.35 T MR scanner with a 6-MV linear accelerator (MR-Linac) [14, 19]. The system allows for the acquisition of three-dimensional (3D) MR scans as well as real-time tumor tracking continuously during treatment delivery by repeated fast planar cine-MRI in a sagittal plane with four frames per second [14].

All patients treated at the MR-Linac were enrolled in an observational study for evaluating feasibility as well as patient acceptance by patient-reported outcomes (PRO). In the current analysis, we describe our institutional experience with the implementation of MRgRT within a high-volume clinical center after 1 year of patient treatments, and review patient-reported outcomes.

## Materials and methods

After obtaining written informed consent, all analyzed patients were included in a prospective observational clinical trial, which had been approved by the local ethics committee. For the period from April 2018 to April 2019, the trial database was interrogated for clinical information, including demographic data, dates of treatment, disease sites treated, dose and fractionation, and treatment duration.

### Simulation and planning

All patients underwent MR simulation directly at the treatment machine. Thereby, not only were MR images for treatment planning generated, but tolerability of immobilization and placement of receiver coils, patient compliance to breathing instructions, and ability to perform inspiration breath-hold were also assessed. Depending on the treatment region, 3D simulation MR images were acquired either in free breathing or in inspiration breath-hold, followed by planar cine-MRI in a sagittal plane to evaluate target motion characteristics. The pulse sequence used for both was always a trueFISP sequence [14], which is the only pulse sequence currently used clinically on the system. The acquisition time of 3D simulation MR images ranged from 17 s in breath-hold to about 3 min for pelvic scans in free breathing, with an in-plane resolution of 1.5 × 1.5 mm^2^ and slice thickness of either 1.5 mm or 3 mm. MR simulation was carried out without administration of contrast agent. Subsequently, native CT simulation scans were performed on the same day for each patient with identical immobilization devices and simulation dummy coils, usually reproducing the same breathing state as in the MR simulation. Additional contrast-enhanced CT scans were acquired if no contraindications were present (e.g., renal dysfunction or allergies against contrast media). For treatment planning, the integrated treatment planning system (TPS) of the MRIdian Linac was used. Following simulation, MRI and CT datasets were transferred to the TPS and deformably registered using the vendor-supplied deformation algorithm, which for multimodal deformable image registration iteratively tries to minimize a dissimilarity measure computed from mutual information. When available, diagnostic MR images were additionally imported and rigidly registered with focus on the region of the GTV. Gross tumor volumes (GTV) comprised the sum of macroscopic tumor delineated in all co-registered modalities. Depending on the region to be treated, clinical target volumes (CTV) were expanded from the GTV using margins of 1 mm (for example pelvic lymph nodes) to 5 mm (for example hepatic metastases). An isotropic planning target volume (PTV) margin of 4 mm was added in order to account for technical inaccuracies.

For all patients, the simulation MRI datasets were chosen as primary image sets for treatment planning, in order to facilitate same-modality image registration during daily treatment. Electron density information for dose calculation was derived from the registered CT scans. Step-and-shoot treatment plans were generated using the dedicated TPS, where dose calculation was always performed via Monte Carlo dose calculation taking into account the static magnetic field.

### Treatment

Daily image guidance was performed for each fraction by onboard 3D MRI using identical settings (field of view, duration, pulse sequence, breathing instructions) as during MR simulation. Soft tissue-based registration with the reference MR scan was applied, usually registering directly on the GTV, and the couch shifted accordingly.

Real-time MR gating was used for all patients for whom respiratory movement of the target was detected during MR simulation. All respiratory-gated treatments were performed in inspiration breath-hold. A gating target (region of interest, ROI) was defined (either the tumor/resection cavity or a surrogate, e.g., vessel, bronchus in close proximity), and the gating boundary was selected as a numerical margin added to the target ranging between 3 and 4 mm at the discretion of the treating physician. Due to uncertainties in deformable registration and image noise possibly causing errors in contour tracking [17], a small percentage of target outside of the predefined boundary without triggering a beam-off event was allowed (threshold-ROI%) [17]. Threshold-ROI% was set to 3% for most of the cases and to values up to 7% in isolated special cases. The system software automatically stopped radiation delivery when this threshold was exceeded. If intrafractional changes in target position were detected in the cine-MR, no two-dimensional table shifts were performed. Instead, the treatment was always interrupted and a repeat volumetric MRI scan was performed to allow a 3D positional correction.

**Fig. 1:**
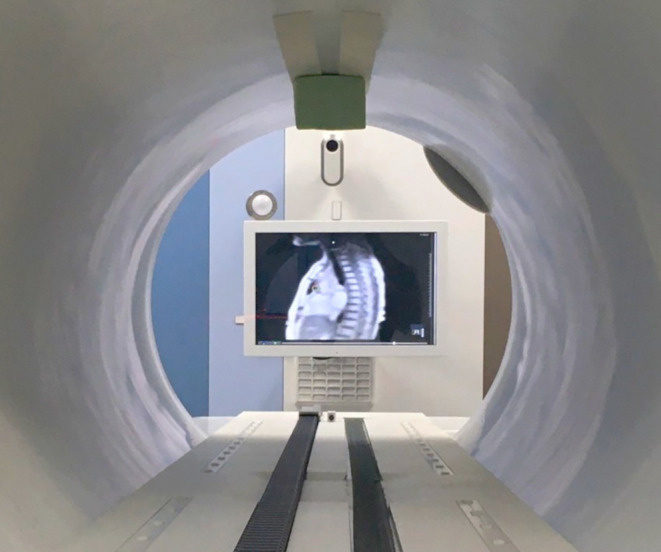
In-room screen used for live visual patient feedback during gated treatments

During gated delivery, patients were provided with visual guidance via an in-room screen displaying the live sagittal cine-MR image with an overlay of gating target and boundary, thus enabling them to steer their repeated breath-holds to the right position. This idea was adopted from other published in-room screen solutions for MRgRT [17, 20]. Patients were able to observe this monitor during the whole treatment with the help of a mirror fixed to the bore (see Fig. 1). If necessary, patients received assisting breathing commands in order to find the optimal breath-hold. In addition to respiratory gating, the gating functionality of the system was also used for target tracking on patients in whom the target did not move with respiration, in order to ensure that no other movements happened during treatment [21].

### Design of the patient-reported outcome questionnaire

Patient-reported acceptance of the whole treatment procedure was documented using an in-house developed patient-reported outcome questionnaire (PRO‑Q; see Table 1), which was completed after the first fraction, weekly during the treatment, and after the last fraction. The PRO‑Q consisted of questions regarding potential MR-related experiences and complaints (e.g., noise, bore size, fixation with coils). For patients undergoing respiratory gated treatments with audiovisual feedback, the perception of their active role was additionally evaluated. Items were scored using a five-point scale.

**Table 1:** Patient-reported outcome (PRO) questionnaire

How do you rate …		1	2	3	4	5	
… the treatment at the MRlinac in total?	Very positive						Very negative
… the information provided by the staff before treatment?	Completely sufficient						Totally insufficient
… the friendliness of the staff?	Very friendly						Not friendly at all
… the duration of treatment?	Very short						Extremely long
… the size of the MRI bore?	Very comfortable in size						Extremely narrow
… the positioning during RT?	Very comfortable						Quite uncomfortable
… having to lie still?	Very easy						Quite difficult
… the noise in the MRI?	Very quiet						Very loud
… the temperature in the MRI?	Very warm						Very cold
… the local temperature of your body parts?	Very warm						Very cold
… potential tingling sensations in your fingers and toes?	Did not occur						Occurred very much
… the breathing instructions?	Easy to understand						Very difficult to understand
… holding your breath during RT?	Easy to do						Very difficult to do
Were you anxious during treatment?	Not at all						Very much
*Only for patients with respiratory gated delivery*							
Was it difficult to control the target by holding your breath?	Not at all						Very much
Was it disturbing to watch your tumor on the monitor?	Not at all						Very much
How did you like the possibility to have an active role in controlling the duration of treatment?	Very helpful						Not helpful at all

In addition to the experience reported by the patients, the staff on the system (therapists and physicians) were questioned about overall patient compliance for each patient after the first and last fraction. They were asked to score the overall compliance of the patient with the treatment procedure on a scale from 1 (very uncomplicated) to 10 (very complicated).

### Statistical analysis

Data analysis was performed with the help of Excel 2010 (Microsoft Corporation; Redmond, USA) as well as SPSS (version 20.0; IBM, Armonk, USA). The Wilcoxon signed-rank test was applied for comparing matched samples. Significance was noted for *p*-values of ≤0.05.

## Results

### Patient and treatment characteristics

From April 2018 to April 2019, 43 patients were treated on the MRIdian Linac system, with a total of 428 fractions. Patients had a mean age of 64 years (range 32–87) at the beginning of treatment, were mainly male (58%), and had a median Karnofsky performance score of 80%. Patient characteristics and treated tumor sites are illustrated in Table 2. The most frequently treated anatomic sites were abdominal nodal metastases and liver lesions. MRgRT was selected for these patients due to a variety of reasons, including superior soft tissue contrast or gated dose delivery and mostly for a combination of these factors. For five patients with bony lesions, a benefit of soft tissue contrast was assumed because the lesions were only limitedly visible on CT scans and had been diagnosed with positron-emission tomography (PET) or scintigraphy before. Three patients with centrally located pulmonary lesions were also treated on the MRIdian Linac, as daily MRI facilitated better distinction of the target volumes from the mediastinum. Stereotactic body radiotherapy (SBRT) was applied in 20 patients (47%). Total applied doses ranged from 4 to 66 Gy, with single doses ranging from 2 to 15 Gy. The mean number of fractions per patient was nine (range 2–33). For another 23 patients, MRgRT was initially foreseen but could not be performed due to different reasons (see Table 3).

**Table 2:** Patient and treatment characteristics

	Number	%	Mean	Minimum	Maximum
*Age (years)*	43		64	32	87
*Sex*					
Male	25	58.1	–	–	–
Female	18	41.9	–	–	–
*Pretreatment performance scale (Karnofsky index; %)*	–	–	80	60	90
*Disease sites treated*	48	–	–	–	–
Abdominal lymph node metastases	11	22.9	–	–	–
Liver lesions	8	16.6	–	–	–
Pelvic lymph node metastases	5	10.4	–	–	–
Bone metastases	5	10.4	–	–	–
Adrenal metastases	4	8.2	–	–	–
Mediastinal lymph node metastases	3	6.3	–	–	–
Cardiac tumors	3	6.3	–	–	–
Pulmonary lesions	3	6.3	–	–	–
Prostate cancer	2	4.2	–	–	–
Partial breast	1	2.1	–	–	–
Others	3	6.3	–	–	–
*Single fraction dose (Gy)*	48	–	5	2	15
*Total dose (Gy)*	48	–	37	4	66
*Number of fractions*	48	–	9	2	33

**Table 3:** Screening failures for MRgRT after MR simulation

Reason	Number (*n* = 23)
Poor general condition, breath-hold not sufficient	8
Distant progress evident in simulation MRI	3
No benefit for gating, rotational IMRT superior	3
Newly implanted metal implant or artifacts	2
Claustrophobia	2
Miscellaneous	5

*MRI* magnetic resonance imaging, *IMRT* intensity-modulated radiotherapy

Gating was applied for all patients treated with SBRT (*n* = 20) as well as for eight additional patients. For all patients, the mean treatment time (time from start of acquisition of the first MRI sequence until completion of dose delivery) amounted to 34 min, with a mean beam-on time of 2:17 min. For patients treated with gated radiotherapy, the mean treatment time amounted to 40 min, while a shorter mean treatment time of 24 min was observed if no gating was applied. The mean duty cycle for respiratory gated treatments, defined as the net beam-on time per fraction divided by the overall time when the system was ready to beam during this fraction, was 72%. In about 15% of all fractions, cine-MR during treatment indicated a patient shift which then required repetition of the 3D MR-scan and repositioning of the patient before treatment could be resumed.

### Patient-reported outcomes

Completed questionnaires were available for 34 patients. In total, patients rated MRgRT as positive or at least tolerable, with mean scores of 1.0–3.6 in the 14 main questions (see Table 4). No statistically significant changes were detected between the first fraction and the end of treatment for all assessed questions (*p* ≥ 0.05).

**Table 4:** Results of the patient-reported outcome questionnaires

How do you rate …	After the first fraction (*n* = 34)	At the end of treatment (*n* = 34)	*p*-value
	Mean (range)	Mean (range)	
… the treatment at the MRlinac in total?	1.3 (1–4)	1.4 (1–3)	0.739
… the information provided by the staff before treatment?	1.1 (1–2)	1.1 (1–2)	1.000
… the friendliness of the staff?	1.0 (1–2)	1.0 (1–2)	0.317
… the duration of treatment?	2.2 (2–5)	2.1 (2–4)	0.741
… the size of the MRI bore?	1.9 (1–4)	1.8 (1–4)	1.000
… the positioning during RT?	2.2 (1–4)	2.2 (1–4)	0.604
… having to lie still?	2.0 (1–3)	1.8 (1–4)	0.662
… the noise in the MRI?	2.1 (1–4)	2.0 (1–3)	0.817
… the temperature in the MRI?	3.6 (1–4)	3.4 (1–3)	0.067
… the local temperature of your body parts?	3.5 (1–3)	3.2 (1–4)	0.302
… potential tingling sensations in your fingers and toes?	1.9 (1–4)	1.7 (1–4)	0.090
… the breathing instructions?	1.1 (1–3)	1.2 (1–2)	0.102
… holding your breath during RT?	1.4 (1–3)	1.5 (1–3)	0.305
Were you anxious during treatment?	1.4 (1–3)	1.3 (1–3)	0.157
Respiratory gated dose delivery (*N* = 22)			
Was it difficult to control the target by holding your breath?	1.3 (1–3)	1.2 (1–2)	0.739
Was it confronting to watch your tumor on the monitor?	1.2 (1–2)	1.1 (1–2)	0.564
How did you like the possibility to have an active role in controlling the duration of treatment?	1.2 (1–2)	1.1 (1–2)	1.000

However, several patients (65%) reported some degree of potential MR-related complaints at least once (score ≥4). Patients mainly complained about the temperature in the room (24%) and of some particular body parts (27%). Furthermore, 18% of the patients experienced paresthesia during treatment and 12% rated the positioning as well as having to lie still for at least half an hour during treatment negatively (score ≥4). The size of the MRI bore and the duration of treatment were classified as at least narrow or long, respectively, by 6% of the patients. Despite the routine use of headphones, 3% of the patients scored the noise of the MRI as disturbing.

### Patient experience with audiovisual feedback

The sub-section of the questionnaire related to breath-hold-gated dose delivery with audiovisual feedback was completed by 22 patients. No patient reported severe difficulties in controlling the target by holding his/her breath. Additionally, no patient answered that watching his/her tumor on the monitor during treatment was considerably disturbing. All patients seemed to appreciate their active role in controlling the duration of treatment.

### Staff-rated patient compliance

The mean overall patient compliance with the treatment procedure rated by the staff was 4.1 (range 1–9) after the first fraction and 3.1 (range 1–8) after the last fraction on a scale from 1 (very uncomplicated) to 10 (very complicated) (*p* = 0.17).

### Acute toxicity

Patients tolerated the treatment very well with only mild acute toxicity. In detail, patients reported fatigue CTCAE grade I (*n* = 19), nausea CTCAE grade I (*n* = 12), coughing CTCAE grade I (*n* = 7), flatulence CTCAE grade I (*n* = 6), diarrhea CTCAE grade I (*n* = 4), dyspnea CTCAE grade I (*n* = 2), dysphagia CTCAE grade I (*n* = 2), dyspepsia CTCAE grade I (*n* = 7), and pain in the thoracic wall CTCAE grade I (*n* = 2). Except for 4 patients with fatigue CTCAE grade II, no acute toxicity ≥ CTCAE grade II was detected. 11 patients did not describe any aggravation of pre-existing symptoms or the occurrence of new symptoms after RT.

## Discussion

MRgRT has been successfully introduced into clinical practice at our institution. Thereby, patient-reported outcomes were an adequate tool to assess tolerability of the treatment at the MR-Linac. This is important because aside from the technical difficulties coming along with the integration of medical linear accelerators with MR scanners, the introduction of dedicated devices for MRgRT also implies new challenges for operating staff as well as patients. Patients at the MR-Linac are not only immobilized, but also need to tolerate placement of receiver coils while positioned in the bore for a longer time [22]. It is known that patients can experience claustrophobia and anxiety in an MR scanner [23, 24], and the rate usually increases with longer and narrower bore openings [23, 25]. Due to the split-magnet design [14], the MRIdian Linac has a tunnel length of about 232 cm, which is longer than at contemporary diagnostic MR scanners and could potentially contribute to anxiety [25]. While the absolute numbers of diagnostic MR scans terminated early due to claustrophobia or anxiety are generally small, in the range of 1–2% [24], early termination of fractions in radiation therapy for this reason needs to be prevented as effectively as possible.

In our cohort, all fractions could be administered safely, without patient-induced early terminations, and, as expected, without any treatment-related severe toxicities. The PRO‑Q results shown in this manuscript confirm that treatment at the MR-Linac is generally well tolerated by patients, which is in accordance with results previously published by Tetar et al. [26]. Compared to their rate of MR-related patient complaints of 29%, the value of 65% in our study is considerably higher, while, on the other hand, none of our patients reported considerable anxiety. The fact that despite a 65% complaint rate, mainly about temperature, paresthesia, and immobilization in general, the patients in our study still rated the total experience as at least tolerable, and that no patient reported considerable anxiety, might be due to the increased attention they receive from the MR-Linac staff [27, 28]. MR simulation on the MR-Linac seems to also help in the context of patient anxiety, as it allows patients to get acclimatized with the whole procedure [28]. However, thorough patient screening is still necessary in order to avoid early treatment termination due to patient noncompliance.

A limitation of this study is the relatively small number of patients. Therefore, we will continue to monitor PRO at the MR-Linac. Potentially, this information can also be used to further improve the processes in terms of patient experience [27]. Additional limitations lie in the fact that dedicated diagnostic MRI planning sequences were not available for every patient. Hence, the full potential of MRI for target delineation (particularly with the help of perfusion- or diffusion-weighted images) could not be exploited. Furthermore, diagnostic planning MRI sequences were not acquired in the treatment position, so that the co-registered images were only of limited use for target delineation, and deformable registration would have introduced additional uncertainties. Further studies involving dedicated diagnostic MRI in treatment position for target delineation as well as for follow-up imaging are therefore planned. In addition, future studies need to assess the question of which additional pulse sequences apart from the trueFISP sequence are needed for low-field MRgRT systems and how they compare to 1.5 T-systems [29].

We have shown that MR-guided respiratory gating in breath-hold is feasible and, combined with a real-time audiovisual feedback system, was very well tolerated and appreciated by patients. This confirms the positive results of Tetar et al. [26]. Cine-MR-enabled gating in breath-hold is also effective; we observed a mean gating duty cycle of 72%, similar to the range of 67% to 87% published by van Sörnsen de Koste et al. [17] using a predecessor of the MRIdian Linac system [12]. The gating duty cycle is influenced by the size of the gating boundary as well as by the threshold-ROI% value. Compared to their study, we have used slightly larger gating boundaries of up to 4 mm but, on the other hand, considerably smaller threshold-ROI% values. Apart from the paper by van Sörnsen de Koste et al. [17], a few phantom studies on the accuracy and influencing factors of MR-guided gating using low-field MRgRT systems have been published [30, 31]. However, the impact and different contributing effects still need to be studied using real patient data on a larger scale.

Besides respiratory gating, cine-MR-based structure tracking can also be used to monitor targets that do not move with respiration, for example the prostate. First published results show that this facilitates safe administration of MR-guided ultra-hypofractionated prostate treatments, potentially enabling margin reduction while eliminating the need for fiducial or transponder implantation [32].

A number of authors have reported on on-table adaption of treatment plans using MRgRT devices [11, 33–37] and also on assessing which patients benefit most from adaptive MRgRT [35, 38–44]. In the presented study, no online treatment plan adaptions were performed. Even without online adaption, MRgRT adds distinct value by better target visualization due to improved soft-tissue contrast. There might be a lot of cases where the enhanced soft tissue contrast alone improves the accuracy of radiation therapy compared to standard IGRT techniques. Noel et al. [4] have reported on physician-rated organ at risk and target visualization in onboard MR compared to onboard cone-beam CT, and visualization in MRI was rated better for 71% of all structures. There is a need for further evaluation of this aspect as well as for the opposing scenario, i.e., online adaption of treatment plans using conventional IGRT techniques.

Head-to-head comparative studies of CT-guided and MR-guided adaptive radiotherapy applying standard doses and fractionation might not be sufficient, as MR-guided adaptive radiotherapy allows for high-dose radiotherapy under circumstances in which treatment would not have been possible with conventional techniques [34]. Further well-designed clinical trials will be necessary to fully demonstrate the true potential of MR-guided adaptive radiotherapy. Radiation oncologists are now forced to reconsider the paradigms of total dose determination at the beginning of treatment and equal dose delivery during each single fraction.

Although there are many remaining questions to be answered, MR-guided adaptive radiotherapy offers the chance for tailormade, daily individualized radiotherapy in order to further reduce side-effects in cancer therapy and to improve tumor control and survival.
